# Mechanistic Analysis of Physiological and Metabolic Responses in Non-Jointed Water Dropwort Under Phosphorus Stress

**DOI:** 10.3390/metabo16020101

**Published:** 2026-01-29

**Authors:** Bingqing Lu, Zhengnan Cen, Xiyu Zhang, Ting Xue, Yu Guo

**Affiliations:** 1 School of Biomedicine and Pharmacy, Suzhou Chien-Shiung Institute of Technology, Suzhou 215411, China; 2 Suzhou Chien-Shiung Biopharmaceutical Industry Think Tank, Suzhou 215411, China; 3 Laboratory for Statistical Monitoring and Intelligent Governance of Common Prosperity, Collaborative Innovation Center of Statistical Data Engineering Technology & Application, School of Statistics and Data Science, Zhejiang Gongshang University, Hangzhou 310018, China; 4 Zhejiang Key Laboratory of Solid Waste Pollution Control and Resource Utilization, School of Environmental Sciences and Engineering, Zhejiang Gongshang University, Hangzhou 310018, China; 5 Anhui Agricultural University, Hefei 230036, China

**Keywords:** water dropwort, phosphorus stress, UHPLC-MS, metabolomics

## Abstract

Background: Non-jointed water dropwort (*Oenanthe javanica* (Blume) DC.) is a widely cultivated aquatic vegetable with notable nutritional and pharmacological properties. Phosphorus (P) is a key nutrient affecting plant growth, photosynthesis, and metabolic balance, yet its role in water dropwort remains understudied. Methods: This study investigated the physiological and metabolic responses of non-jointed water dropwort under P-deficiency treatment (0 mg·L^−1^) and increasing P supply levels (5, 10, and 30 mg·L^−1^). Results: Moderate P supply (10 mg·L^−1^) significantly promoted plant growth, enhanced photosynthetic efficiency, and increased antioxidant enzyme activity, increasing by 55.9%, 20.2%, and 118%, respectively, compared with the P-deficiency treatment. High P levels (30 mg·L^−1^) inhibited growth and induced oxidative stress. Untargeted metabolomic analysis was conducted on root samples from CK (0 mg·L^−1^) and HP (30 mg·L^−1^) groups using UHPLC-MS. A total of 1274 metabolites were identified, with flavonoids, phenylpropanoids, fatty acid and conjugates being predominant. A total of 842 differential metabolites were screened under HP stress, with flavonoids (e.g., narcissin) showing the most significant upregulation. KEGG enrichment revealed key pathways including biosynthesis of amino acids, ABC transporters, and aminoacyl-tRNA biosynthesis, indicating metabolic reprogramming under HP stress. Notably, flavonoid and terpenoid pathways were upregulated, while certain lipid metabolism pathways, including fatty acid conjugates and phenylpropanoids, were downregulated. These findings suggest that non-jointed water dropwort adapts to high P stress by activating defense-related secondary metabolism and adjusting carbon–nitrogen allocation. Conclusions: This study provides a theoretical basis for P management and stress-resistant cultivar selection in non-jointed water dropwort.

## 1. Introduction

Water dropwort (*Oenanthe javanica* (Blume) DC.) is a perennial aquatic herbaceous plant of the Apiaceae family, widely distributed across East and Southeast Asia [1]. In China, it is traditionally used both as a vegetable and as a medicinal herb, rich in proteins, dietary fiber, flavonoids, and other bioactive compounds. These components confer a range of pharmacological effects, including antioxidant, anti-inflammatory, neuroprotective, anticancer, cardioprotective and analgesic activities [2,3,4]. Through long-term cultivation, China has developed over 140 cultivars. Among them, “Jintan Non-jointed Water Dropwort”, a cultivar distinguished by its lack of jointed stems, is highly valued for its high yield, quality, stress tolerance, and export potential [5]. Recent studies have mainly focused on breeding and cultivation techniques to improve resource efficiency and industrial output [6]. However, its physiological responses under nutrient stress, especially phosphorus (P) deficiency, are still not well understood.

P plays an irreplaceable role in plant growth by participating in energy transfer, nucleic acid synthesis, and membrane structure stabilization [7]. However, P availability in soil is often limited because inorganic phosphate is easily fixed into insoluble compounds, making it largely inaccessible to plants. P stress subsequently suppresses plant growth, hinders root development, and compromises crop yield and quality [8]. To cope with P stress, plants adopt various strategies such as modifying root architecture, activating signaling pathways, and adjusting osmotic and antioxidant systems [9]. P availability also profoundly influences photosynthetic efficiency and redox homeostasis. Low P impairs chloroplast development, promotes reactive oxygen species (ROS) accumulation, and triggers the activation of antioxidant enzymes to mitigate oxidative damage [10]. On a metabolic level, P deficiency reshapes primary and secondary metabolic networks by regulating sugar, organic acid, amino acid, flavonoid, and phenylpropanoid pathways, thereby optimizing internal resource allocation and enhancing stress tolerance [11,12,13]. In many crop species, typical responses to P stress include enhanced acid phosphatase activity, increased organic acid exudation, and the accumulation of stress-related metabolites [14]. Therefore, investigating plant responses across a gradient of P availability—from deficiency to excess—is essential for improving P use efficiency and breeding resilient cultivars, ultimately enabling precision fertilization [15,16]. Hence, understanding plant responses to P stress is vital for improving P-use efficiency and breeding resilient cultivars, ultimately enabling precision fertilization.

Although studies on P stress in water dropwort are scarce, the species has demonstrated strong adaptability to various abiotic stresses such as heavy metal, salt, and drought stress. Under Cd, Pb, and As exposure, water dropwort enhances the activity of antioxidant enzymes (SOD, POD, CAT) to alleviate oxidative damage, with chlorophyll and soluble sugar contents showing concentration-dependent changes [17,18]. In response to salt stress, it accumulates proline, soluble sugars, and polyphenolic compounds while activating relevant antioxidant pathways and stress-responsive genes [4,19]. Drought conditions have been shown to increase total phenolic content and antioxidant capacity, indicating a degree of drought tolerance [20,21]. In addition, nutrient solution studies and metabolomic analyses have revealed significant changes in secondary metabolites under different growth stages and stress conditions [22,23,24]. These findings provide indirect evidence that water dropwort may also employ metabolic and physiological adjustments to cope with P stress, though direct studies on this topic are lacking.

For non-jointed water dropwort cultivars, only one study has examined the correlation between Pb content in irrigation water and its accumulation in stems [25]. To date, no research has addressed its growth, physiological, or metabolic responses to P stress. This lack of information limits our understanding of its P nutrition adaptability and hinders the development of science-based cultivation and nutrient management strategies. Therefore, this study conducted a systematic investigation into the effects of different P levels on the growth, physiological traits, and metabolic profiles of non-jointed water dropwort. Defining its optimal P supply range will provide a scientific foundation for precision P management, high-quality yield production, and environmentally sustainable cultivation.

## 2. Materials and Methods

### 2.1. Plant Material and Growth Conditions

This study used the local cultivar “Jintan Non-jointed Water Dropwort” from Changzhou, Jiangsu Province. The experiment started in April 2024 and took place in a vegetable experiment greenhouse. During the experiment, the greenhouse was maintained at a temperature of 30/25 °C, with a diurnal cycle of 16/8 h, an approximately light intensity of 300 µmol m^−2^ s^−1^, and a relative humidity of 70%. On 5 April, pretreated seeds were evenly sown in commercial seedling substrate (pH 6.5, EC 1.2 mS/cm). Seeds germinated approximately two weeks after sowing. When seedlings reached the three-leaf stage, uniformly, healthy plants (~10 cm tall) were selected for transplanting. Each growth tray (60 cm × 30 cm × 13 cm) contained 16 plants of water dropwort. All plants were managed using standard hydroponic cultivation practices with consistent water levels.

### 2.2. Experimental Design and P Treatment

The base nutrient solution was prepared using a commercial water-soluble fertilizer (“Huaduoduo No. 1”, EC 0.8 mS·cm^−1^), which contains 20% total nitrogen (including nitrate-N, ammonium-N, and urea-N), 20% P_2_O_5_, and 20% K_2_O, along with trace elements (B, Cu, Fe, Zn, Mo, and Cr) as specified by the manufacturer. Sodium dihydrogen phosphate served as the external phosphorus (P) source. Based on previous studies on soybean and Paspalum notatum [15,16], we designed four P treatments to simulate a range of P stress levels: (1) CK (0 mg/L), (2) LP (low P, 5 mg/L), (3) MP (moderate P, 10 mg/L) and HP (high P, 30 mg/L). These concentrations are within the common range used in nutrient solution studies of aquatic and semi-aquatic plants. The hydroponic system was based on a solid substrate cultivation method, using peat and vermiculite (3:1, *v*/*v*) in seedling trays with a constant nutrient solution level maintained to ensure root immersion. The initial pH of the nutrient solution was approximately 6.5, and no pH adjustment was made after P addition to simulate realistic cultivation conditions.

For each treatment, the required amount of NaH_2_PO_4_ was dissolved in distilled water, and 1 L of fresh nutrient solution was used to completely replace the existing solution in each tray every 10 days. This replacement was repeated four times over a 40-day experimental period to maintain stable P concentrations. Each treatment included three replicates, with 30 uniform seedlings per replicate. All other nutrients and environmental conditions remained identical across treatments to ensure data consistency. At the end of the treatment period, we randomly selected 20 uniform plants from each group for physiological measurements.

To further examine the metabolic response of water dropwort roots to P stress, we performed untargeted metabolomic analysis under CK and HP conditions, focusing on small-molecule metabolites rather than peptides or proteins. After 40 days, we randomly selected three vigorous and morphologically similar plants from each treatment. We harvested the root tissues, rinsed them with distilled water to remove residual substrate, gently blotted them dry, and immediately froze them in liquid nitrogen. Under liquid nitrogen, we ground the samples into fine powder using pre-chilled mortars and pestles. This process yielded six powdered samples (three per treatment), which we stored at −80 °C until analysis. Beijing Tsingke Biotech Co., Ltd. (Suzhou, China) performed the metabolomic profiling using ultra-high-performance liquid chromatography–mass spectrometry (UHPLC-MS), including metabolite extraction, separation, identification, and quantification to ensure comprehensive and high-accuracy results.

### 2.3. Growth and Physiological Measurements

To comprehensively evaluate the effects of different P levels on the growth and photosynthetic characteristics of water dropwort, a completely randomized design was applied. Each treatment included five biological replicates. Growth parameters included plant height, stem diameter, leaf length and width, number of effective leaves, number of tillers, leaf area, and both fresh and dry biomass of shoots and roots. Dry weight was determined after oven-drying at 105 °C for 30 min, followed by drying at 80 °C to constant weight. The root-to-shoot ratio based on dry weight was calculated to assess biomass.

Photosynthetic pigments, including chlorophyll a, chlorophyll b, total chlorophyll, and carotenoids, were extracted with 95% ethanol [4]. Samples were ground with quartz sand and anhydrous sodium sulfate, filtered, and brought to volume in brown flasks. After standing in the dark for 24 h and centrifugation, absorbance was measured at 665, 649, and 470 nm using a UV-1900i spectrophotometer (Shimadzu, Kyoto, Japan). Gas-exchange parameters, such as net photosynthetic rate (Pn), stomatal conductance (Gs), intercellular CO_2_ concentration (Ci), and transpiration rate (Tr), were measured using a LI-6400XT portable photosynthesis system (LI-COR, Lincoln, NE, USA). Measurements were conducted under a light intensity of 1200 μmol·m^−2^·s^−1^ and a CO_2_ concentration of 400 μmol·mol^−1^ to ensure accuracy and consistency.

To assess oxidative stress responses, the activities of key antioxidant enzymes—superoxide dismutase (SOD), catalase (CAT), and peroxidase (POD)—were measured. In addition, malondialdehyde (MDA) content was determined to evaluate lipid peroxidation levels. All assays were conducted using commercial kits (SOD-2-W, CAT-2-W, POD-2-Y, MDA-2-Y) from Suzhou Keming Biotechnology Co., Ltd. (Suzhou, China) [26,27].

Nutritional quality was assessed by measuring soluble sugar, ascorbic acid (AsA), soluble protein, flavonoids, and total phenolics. Soluble sugar and AsA levels were determined using commercial kits (KT-2-Y and ASA-2-W). Soluble protein was quantified with the Coomassie brilliant blue G-250 method [28]. Flavonoid content was measured by aluminum chloride colorimetry using rutin as the standard [23]. Total phenolics were determined with the Folin–Ciocalteu method against a gallic acid standard curve [29].

### 2.4. Metabolomic Analysis

For metabolomic analysis, the whole root system was extracted with a methanol–acetonitrile–water solution (2:2:1, *v*/*v*/*v*) containing isotope-labeled internal standards. Metabolite profiling was performed using a Vanquish UHPLC system (Thermo Fisher Scientific, Waltham, MA, USA) coupled with an Orbitrap Exploris 120 high-resolution mass spectrometer (Thermo Fisher Scientific). Data were acquired in both positive and negative ionization modes. Key MS parameters included: a full scan resolution of 60,000, MS/MS resolution of 15,000, stepped collision energies of 20/30/40, spray voltage of ±3.6 to 3.8 kV, and a capillary temperature of 320 °C. Quality control (QC) samples were analyzed regularly throughout the sequence to ensure data accuracy and instrument stability.

### 2.5. Data Analysis

Metabolomic data were preprocessed through outlier removal, missing value imputation, and normalization using isotope-labeled internal standards. Total ion chromatograms (TICs) were used to assess data quality. Metabolites were identified using R-based metabolomics analysis tools and annotated against the BiotreeDB (v3.0) and the plant-specific BT-Plant (v1.1) databases to ensure annotation accuracy [30].

Metabolomic data are characterized by high dimensionality and small sample size, requiring multivariate statistical approaches to identify biologically meaningful differential metabolites [31,32]. First, principal component analysis (PCA) was used to reduce data dimensionality and visualize sample distribution trends [33]. Models were constructed using SIMCA software (V18.0.1), with key parameters such as R^2^X, A, and N reported. To identify differential metabolites, orthogonal partial least squares discriminant analysis (OPLS-DA) was applied. Model reliability was evaluated by cross-validation and 200-time permutation testing. Differential metabolites were selected based on VIP > 1 and *p* < 0.05, ensuring statistical and biological relevance. Hierarchical cluster analysis (HCA) and heatmaps visualized expression patterns between HP and CK groups.

To further investigate the metabolic response of water dropwort under P stress, Kyoto Encyclopedia of Genes and Genomes (KEGG) annotation and enrichment analysis were performed on the identified differential metabolites [34]. By mapping these metabolites to specific pathways, related biological processes were revealed. Enrichment factors were used to evaluate the relative significance of each pathway, with higher values indicating greater pathway involvement in the stress response [35].

## 3. Results

### 3.1. Growth and Physiological Responses to Phosphorus Stress

#### 3.1.1. Changes in Growth Parameters

Non-jointed water dropwort was subjected to four phosphorus (P) treatments: no P (CK), low P (LP), moderate P (MP), and high P (HP). This design aimed to systematically assess the effects of P levels on key growth parameters (Table 1). The results showed that plant height, stem diameter, leaf size, leaf area, and tiller number reached the highest values in MP, followed by a significant decline in HP. It presents a typical “promotion at low level and inhibition at high level” pattern. Plant height in MP reached 38.78 cm, significantly higher than in CK and LP, while HP showed the lowest value (20.05 cm). Stem diameter and leaf area were also maximized in MP (6.32 mm and 1.38 cm^2^), significantly outperforming other treatments. MP treatment markedly promoted tillering and leaf expansion. Visual differences among treatments were also evident in plant morphology, as shown in Figure S1. In terms of biomass accumulation, the MP group showed the highest shoot fresh and dry weights (70.55 g and 9.87 g), and root fresh and dry weights were also significantly elevated. In contrast, HP treatment led to substantial reductions in both fresh and dry biomass and lowered the root-to-shoot ratio to 5.08. Overall, moderate P supply facilitated vegetative growth and structural biomass accumulation, while excessive P stress suppressed growth and disturbed resource allocation within the plant.

#### 3.1.2. Changes in Photosynthetic Parameters

To investigate P effects on photosynthesis, pigment content and photosynthetic parameters were measured (Table 2). P levels significantly affected chlorophyll a, chlorophyll b, total chlorophyll, and carotenoid contents (*p* < 0.05), showing a pattern of stimulation at moderate levels and inhibition at high levels. The MP treatment showed the highest chlorophyll a (1.75 mg·g^−1^), chlorophyll b (0.76 mg·g^−1^), and total chlorophyll contents (2.51 mg·g^−1^). In contrast, HP exhibited the lowest pigment levels, indicating that excessive P suppressed pigment synthesis.

The chlorophyll a/b ratio did not differ significantly among treatments, suggesting that P mainly affected total pigment accumulation rather than photosystem structure. Carotenoid content also peaked in MP (0.46 mg·g^−1^). Moderate P also increased net photosynthetic rate (10.13 μmol·m^−2^·s^−1^) and stomatal conductance (0.68 mol·m^−2^·s^−1^), while both parameters decreased significantly under HP stress. Intercellular CO_2_ concentration rose sharply under HP stress (358.95 μmol·mol^−1^). Transpiration rate was highest in MP levels (4.27 mmol·m^−1^·s^−1^) and lowest in HP.

#### 3.1.3. Changes in Antioxidant Capacity

**Figure 1:**
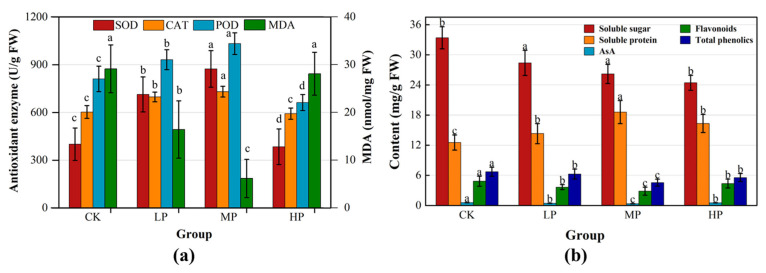
Effects of P levels on antioxidant responses and nutrient composition of non-jointed water dropwort: (**a**) antioxidant enzyme activities (left Y-axis) and MDA content (right Y-axis); (**b**) nutrient composition, including soluble sugar, soluble protein, ascorbic acid, flavonoids, and total phenolics. Data are presented as mean ± standard error. Error bars represent SE from three biological replicates. Different lowercase letters indicate significant differences at *p* < 0.05.

P stress significantly influenced the antioxidant defense system of non-jointed water dropwort (Figure 1a). Activities of three major antioxidant enzymes (SOD, CAT, POD) showed a rise-then-decline trend with increasing P levels. The MP group showed the highest activities, which were significantly higher than those in other treatments (*p* < 0.05), with SOD, CAT, and POD reaching 874, 731, and 1033 U·g^−1^, respectively. In contrast, enzyme levels in HP declined significantly, with SOD dropping to 385 U·g^−1^. Malondialdehyde (MDA) content was also significantly affected by P treatment. The MP group had the lowest MDA level (6.16 nmol mg^−1^), significantly lower than CK (29.11 nmol mg^−1^) and HP (28.09 nmol mg^−1^), while no significant difference was observed between CK and HP.

#### 3.1.4. Changes in Nutrient Composition

P levels significantly affected nutrient accumulation in non-jointed water dropwort (Figure 1b). In carbon metabolism, LP and MP groups had higher soluble sugar contents (28.41 and 26.21 mg·g^−1^), significantly above the HP group. In nitrogen metabolism, soluble protein content peaked in MP (18.61 mg·g^−1^), with the lowest level in CK (12.55 mg·g^−1^). Protein content showed an initial increase and then decline.

For antioxidant-related secondary metabolites, AsA content was highest in CK (0.58 mg·g^−1^) and lowest in MP (0.37 mg·g^−1^). Flavonoids and total phenols showed similar trends. Their levels were highest in CK and decreased significantly in MP. Conversely, reduced oxidative pressure lowers secondary antioxidant metabolite synthesis in MP. In summary, moderate P improves protein accumulation, maintains C-N metabolic stability, and reduces secondary antioxidant burden, supporting improved nutritional quality.

### 3.2. Root Metabolic Responses to Phosphorus Stress

#### 3.2.1. Qualitative and Quantitative Features of Root Metabolites

UHPLC-MS combined with untargeted metabolomics was used to analyze root samples under CK and HP treatments, with metabolite annotation primarily targeting low-molecular-weight compounds. Figure S2 shows TIC profiles under POS and NEG modes, revealing rich and distinct metabolic features in CK and HP root samples. A total of 54,266 metabolic features were detected in positive and negative ion modes. After MS/MS annotation, 1274 metabolites were identified.

**Figure 2:**
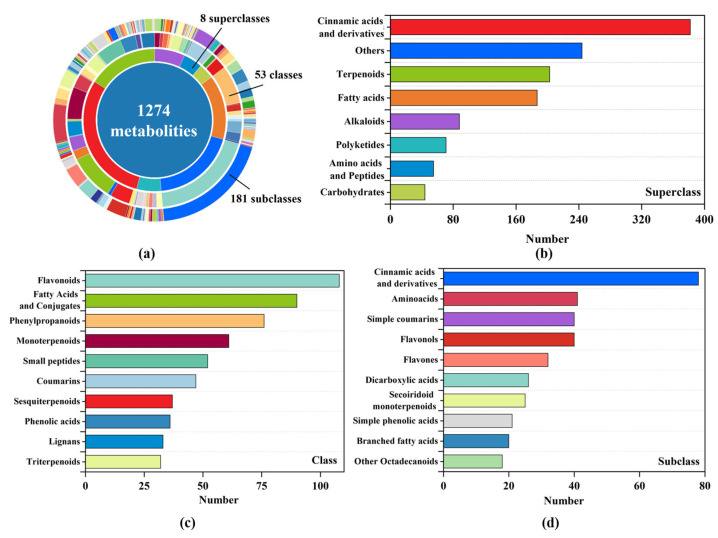
Classification of identified metabolites in non-jointed water dropwort roots: (**a**) multi-level classification of 1274 metabolites; (**b**) number of metabolites in each superclass; (**c**) top 10 metabolite classes by number; (**d**) top 10 metabolite subclasses by number.

These metabolites were classified into 8 superclasses, 53 classes, and 194 subclasses (Figure 2a). Shikimates and phenylpropanoids were the most abundant superclass (382 metabolites, 29.98%). Others (19.15%) and terpenoids (15.93%) followed, suggesting multiple secondary metabolic pathways were activated (Figure 2b). At the class level, flavonoids (108), fatty acids and derivatives (90), and phenylpropanoids (76) were dominant. This indicates enhanced lipid and signaling-related metabolism under high-P stress (Figure 2c). At the subclass level, cinnamic acid derivatives (78) were the most enriched, involved in lignin and flavonoid biosynthesis. Amino acids, simple coumarins and flavonols subclasses also showed significant accumulation (Figure 2d).

#### 3.2.2. Identification and Multivariate Analysis of Differential Metabolites

**Figure 3:**
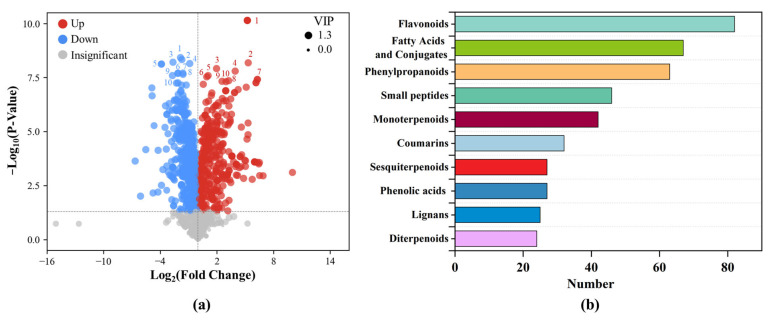
Differential metabolite analysis of non-jointed water dropwort under high phosphorus stress: (**a**) volcano plot showing upregulated and downregulated metabolites between CK and HP groups; (**b**) top 10 enriched classes of differential metabolites based on compound class.

Multivariate statistical methods were used to identify key differential metabolites in roots under HP stress. PCA and OPLS-DA models revealed clear separation between CK and HP groups with good within-group consistency (Figure S3). Permutation tests (n = 200) confirmed model reliability and excluded overfitting, indicating strong stability and predictive power (Figure S4). Based on the screening criteria of VIP > 1 and *p* < 0.05, a total of 842 differential metabolites were identified, accounting for 66.1% of all annotated metabolites. Among them, 475 were upregulated and 367 downregulated under HP stress. As shown in Figure 3a, up- and downregulated metabolites were distinctly distributed between groups. Metabolites with higher VIP values contributed significantly to group discrimination. At the class level, flavonoids were the most abundant (82 compounds, 12.5%). Fatty Acids and conjugates (67), and phenylpropanoids (63) followed, relating to membrane regulation and aromatic metabolism. Other differential metabolites included small peptides (46), monoterpenoids (42), coumarins and phenolic acids (Figure 3b).

**Figure 4:**
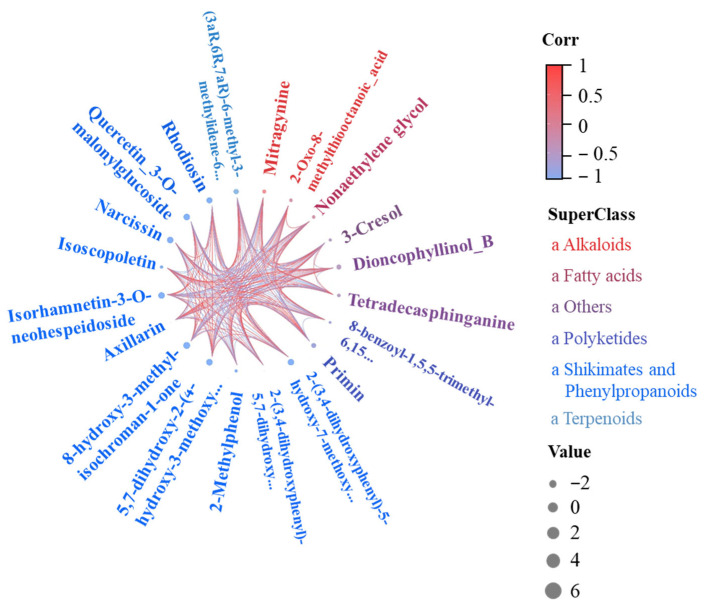
Chord diagram of Spearman correlation for the 10 differential metabolites.

To identify core metabolites, 20 significantly changed compounds were selected as biomarkers (Table S1). Typical upregulated metabolites included narcissin, quercetin-3-O-rutinoside, and 8-hydroxy-3-methyl-isochromenone-1-one. These showed high VIP values and fold changes, suggesting roles in antioxidation, signaling, or pathogen defense. In contrast, mitragynine and tetradecasphinganine were markedly downregulated. To explore regulatory patterns, a Spearman correlation chord diagram was constructed for the top 10 metabolites (Figure 4). Flavonoids formed a strong co-expression module with maximal bandwidth (r = 1).

#### 3.2.3. KEGG Annotation and Enrichment Analysis of Differential Metabolites

**Figure 5:**
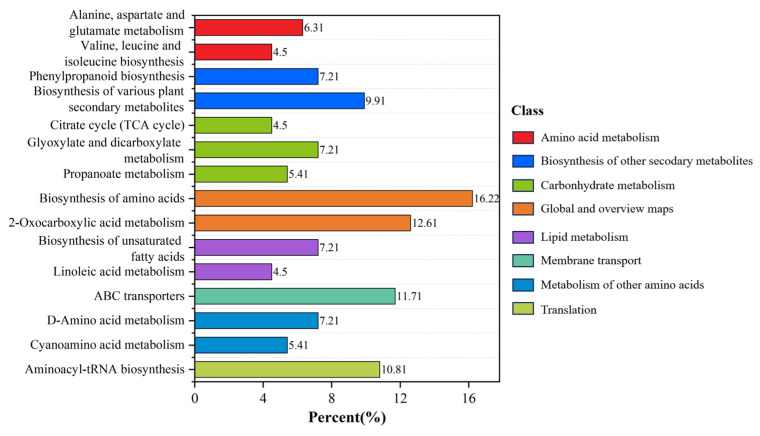
Annotation diagram of a differential metabolic pathway.

To further elucidate metabolic regulation in roots under HP stress, 842 differential metabolites were annotated and enriched using the KEGG database. A total of 89 metabolic pathways were identified, covering primary metabolism, amino acid metabolism, lipid metabolism, and multiple secondary pathways (Figure 5). Among them, biosynthesis of amino acids was the most enriched, accounting for 16.22%. Pathways involved in the biosynthesis of various plant secondary metabolites were also significantly enriched. Several carbon metabolism pathways, such as the citrate cycle (TCA cycle), showed enrichment, reflecting carbon flux reprogramming under P stress. In lipid metabolism, pathways such as the biosynthesis of unsaturated fatty acids were involved. At the transport level, the ABC transporter pathway showed significant enrichment (11.71%), in which thirteen metabolites were enriched (*p* = 1.00 × 10^−5^).

**Figure 6:**
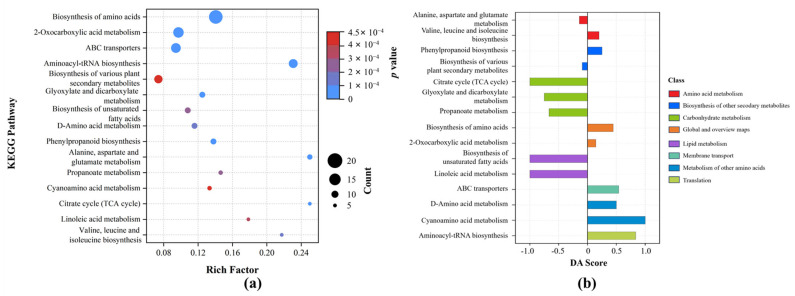
KEGG pathway analysis of differential metabolites in non-jointed water dropwort under high phosphorus stress: (**a**) KEGG enrichment bubble plot showing significantly enriched pathways; (**b**) differential abundance (DA) score plot illustrating up- and downregulated pathways.

KEGG enrichment analysis (Figure 6a) further showed that biosynthesis of amino acids was the most significantly enriched pathway. This pathway contained the highest number of metabolites (18 compounds) and the lowest *p* value (2.52 × 10^−10^), representing a core metabolic pathway in response to HP stress. Other significantly enriched pathways included 2-oxocarboxylic acid metabolism, aminoacyl-tRNA biosynthesis, and ABC transporters. DA score analysis further revealed pathway-level expression trends (Figure 6b). Most pathways showed an overall upregulated tendency, including aminoacyl-tRNA biosynthesis (0.83), ABC transporters (0.54), and D-amino acid metabolism (0.50). In contrast, carbon metabolism pathways such as the TCA cycle (−1) and propanoate metabolism (−0.67), as well as the biosynthesis of unsaturated fatty acids, were predominantly downregulated. Notably, cyanoamino acid metabolism showed a DA score of 1 despite low enrichment, suggesting a potential role in specialized nitrogen regulation.

## 4. Discussion

### 4.1. Growth and Physiological Responses to Phosphorus Levels

Phosphorus (P) is an essential macronutrient involved in energy metabolism, photosynthesis, and membrane lipid synthesis, whose availability directly affects plant growth and stress resistance [7]. In this study, non-jointed water dropwort showed high sensitivity to P levels, exhibiting a “low-promoting, high-inhibiting” response. Moderate P (10 mg·L^−1^, MP) significantly improved plant growth, photosynthetic efficiency, and metabolic balance. In contrast, high P stress (30 mg·L^−1^, HP) suppressed growth and induced oxidative damage and metabolic disorder.

In terms of morphology, plant height, stem diameter, leaf area, and tiller number were highest in the MP group. Root-to-shoot ratio also increased, suggesting that moderate P promotes coordinated shoot and root growth, facilitating nutrient and water uptake. HP treatment significantly inhibited morphological development, possibly due to hormonal imbalance or inhibited cell division. This is consistent with findings in other crops [36,37]. Photosynthetic pigments and performance are key indicators of photosynthetic potential [38]. Chlorophyll a, b, and carotenoid contents increased significantly under MP treatment, enhancing photoprotection and stress tolerance, consistent with previous reports [2,39]. Net photosynthetic rate and stomatal conductance also improved, while intercellular CO_2_ concentration decreased. In contrast, Intercellular CO_2_ concentration rose sharply under HP, suggesting reduced CO_2_ fixation efficiency [40]. This “promotion under moderate P and suppression under high P” trend has also been observed in terrestrial crops such as soybean, maize, and wheat, but non-jointed water dropwort showed particularly high responsiveness in both above- and below-ground traits [11,12,41]. This may reflect a specific adaptation strategy of aquatic vegetables under nutrient fluctuation. Both P deficiency and excess can suppress PSII activity and carbon fixation, leading to reduced photosynthesis [42,43]. In cotton and other species, P deficiency has been linked to reduced photosynthetic rate and ROS accumulation [44].

Antioxidant capacity also responded significantly to P. MP-treated plants had the highest SOD, CAT, and POD and the lowest MDA content. This implies moderate P enhances antioxidant enzyme activity and reduces MDA accumulation, helping to maintain cellular homeostasis under stress. Conversely, enzyme activities decreased under HP and CK treatment, while MDA increased. This suggests both P deficiency and excess disrupt metabolic balance and energy distribution, weakening ROS defense. Similar trends have been reported under nutrient stress in other plants [33,45]. In terms of nutrient accumulation, soluble protein content exhibited an initial increase followed by a decline, with the highest level observed in the MP group, indicating that moderate P facilitates nitrogen assimilation and protein synthesis. Soluble sugar decreased with increasing P. This suggests that moderate P enhances nitrogen metabolism and protein synthesis, while under P deficiency, sugars may support osmotic adjustment and stress protection. Secondary metabolites such as flavonoids and total phenols, along with AsA, were higher in CK and decreased significantly under moderate P, implying that P stress enhances the synthesis of non-enzymatic antioxidants, while moderate P reduces the reliance on such defensive compounds. These findings collectively indicate a shift towards secondary metabolism and antioxidant defense under low P [46].

### 4.2. Root Metabolic Responses to Phosphorus Stress

Under abiotic stress, plants often reprogram metabolic pathways to optimize resource allocation and maintain homeostasis. Metabolic regulation is therefore a key mechanism of stress resistance [47]. Metabolomics enables comprehensive detection of small-molecule dynamics and is effective for elucidating stress responses [48]. UHPLC-MS–based non-targeted metabolomics has been applied in tomato [49], cucumber [50], and wheat [51], but rarely in non-jointed water dropwort. In this study, 1274 metabolites were qualitatively identified in roots. Root tissues were selected as the focal point because roots are the primary site of P uptake and initial stress perception, making them highly responsive to changes in external P availability.

Metabolite classification showed that shikimates and phenylpropanoids were the most abundant, suggesting activation of aromatic metabolism in HP. At the subclass level, flavonoids were particularly prominent, underscoring their central role in the phosphorus stress response [23]. Phenylpropanoids are key precursors of flavonoids, lignin, and coumarins. These compounds enhance cell wall stability and antioxidant capacity under stress [52]. Other classes, fatty acids, and alkaloids were also abundant, indicating activation of multiple secondary pathways [53]. Additionally, the enrichment of amino acids and peptides suggests a regulatory shift in amino acid metabolism, likely supporting osmotic adjustment and stress signaling—a response consistent with previously reported low-phosphorus adaptations in other plant species [33,54].

A total of 842 differential metabolites were identified, including 475 up-regulated and 367 down-regulated compounds, indicating strong metabolic disturbance under high P. Among the altered metabolites, flavonoids were the most abundant class, reinforcing their central role in P stress response and antioxidant defense [24,55]. Up-regulated flavonoids, such as narcissin, suggest activation of flavonoid biosynthesis under P stress [56]. Terpenoids and coumarins were also up-regulated, reflecting coordinated defensive metabolism. In contrast, membrane lipid-related metabolites, including tetradecylsphingosine, were down-regulated under HP stress, consistent with the membrane lipid remodeling model [57]. This pattern, along with decreased levels of specific alkaloids and hydroxylated flavones, suggests coordinated shifts in sphingolipid biosynthesis and nitrogen metabolism that may impair membrane stability. Correlation analysis showed strong negative relationships between flavonoids and lipid/phenolic acids. This indicates competitive resource allocation, similar to patterns in rice and Arabidopsis [58]. Compared to studies in soybean and wheat, where low P often triggers carbohydrate and organic acid metabolism, our findings show that high P in non-jointed water dropwort preferentially activated amino acid and secondary metabolism, suggesting a different reallocation strategy under excess nutrient conditions [12,46].

KEGG enrichment identified 89 pathways, with amino acid metabolism being most prominent. Pathways such as biosynthesis of amino acids are closely linked to nitrogen metabolism, osmotic regulation, and carbon–nitrogen backbone adjustment, which may help maintain osmotic balance and provide energy compensation under stress [59,60]. Enrichment in lipid metabolism pathways, such as biosynthesis of unsaturated fatty acids, suggests that high P supply affects fatty acid saturation and membrane structure regulation, consistent with the classic “P imbalance–membrane lipid remodeling” mechanism [61]. Aminoacyl-tRNA biosynthesis was significantly enriched, indicating enhanced protein translation under stress. This may support synthesis of stress-related enzymes and structural proteins. ABC transporter pathways, often involved in hormone transport and secondary metabolite trafficking, were also enriched. Their upregulation suggests an activation of membrane-mediated signaling and detoxification processes to mitigate high-P-induced stress [62]. Notably, cyanoamino acid metabolism showed enrichment, with most related metabolites up-regulated, which may reflect the plant’s efforts to adjust nitrogen distribution and initiate defense-related signaling cascades under stress [63].

Similar to the responses under heavy metal and salt stress, high P stress in non-jointed water dropwort also triggered enhanced antioxidant metabolism and the accumulation of secondary metabolites, particularly flavonoids and phenolic compounds [4,17]. However, unlike heavy metal stress, where antioxidant enzyme activity often shows a biphasic pattern, P-induced oxidative stress led to a more consistent decline in enzyme activities under high concentrations. Furthermore, while salt and metal stresses typically cause broad-spectrum lipid and osmolyte alterations [19]. In contrast, P stress caused more targeted changes, including downregulation of membrane lipid-related metabolites and specific reprogramming of amino acid pathways.

## 5. Conclusions

This study systematically investigated the physiological traits and metabolic responses of non-jointed water dropwort under different phosphorus (P) levels (0, 5, 10, and 30 mg·L^−1^). The results demonstrated a “low-promoting, high-inhibiting” trend in response to P availability. Moderate P supply (10 mg·L^−1^) significantly enhanced plant growth, biomass accumulation, photosynthetic efficiency, and antioxidant enzyme activity, while maintaining metabolic balance. High P treatment (30 mg·L^−1^), on the other hand, induced oxidative stress and disrupted metabolic homeostasis. A total of 1274 root metabolites were identified, with 842 showing significant changes under HP stress. Metabolomic analysis revealed strong activation of flavonoid, terpenoid, and coumarin pathways, alongside a decline in lipid-related and aromatic metabolites. Enrichment in amino acid metabolism, aminoacyl-tRNA biosynthesis, and cyanoamino acid metabolism pathways suggests that water dropwort adapts to P excess by reallocating nitrogen resources, restructuring membranes, and enhancing antioxidant defense.

In summary, moderate P optimizes physiological performance and nutrient use efficiency, whereas high P induces complex metabolic reprogramming. These findings provide new insights into the adaptive mechanisms of non-jointed water under P stress and offer a theoretical basis for precise P management and breeding of stress-resilient cultivars. Future studies should integrate transcriptomic and proteomic analyses to further elucidate the regulatory networks underlying these responses and directly quantify ROS levels to complement the assessment of oxidative stress.

## Figures and Tables

**Table 1:** Effects of different P levels on the growth parameters of non-joint water dropwort.

Parameter	CK	LP	MP	HP
Plant height/cm	24.87 ± 1.58 c ^1^	27.88 ± 1.03 b	38.78 ± 2.42 a	20.05 ± 1.32 d
Stem diameter/mm	4.57 ± 0.24 c	5.52 ± 0.49 b	6.32 ± 0.45 a	4.03 ± 0.20 d
Leaf length/cm	6.33 ± 0.81 b	6.87 ± 0.65 ab	7.45 ± 0.42 a	5.20 ± 0.48 c
Leaf width/cm	4.13 ± 0.27 c	4.98 ± 0.17 b	5.88 ± 0.42 a	3.47 ± 0.34 d
Leaf area/cm^2^	0.60 ± 0.08 c	1.02 ± 0.12 b	1.38 ± 0.08 a	0.44 ± 0.07 d
Number of leaves/leaves	13.83 ± 2.14 a	14.67 ± 1.51 a	15.83 ± 1.83 a	11.00 ± 1.67 b
Number of tillers/pcs	13.67 ± 1.75 b	16.83 ± 1.47 a	18.67 ± 1.75 a	12.50 ± 1.64 b
Shoot fresh weight/g	58.98 ± 1.79 c	63.13 ± 1.42 b	70.55 ± 1.50 a	49.10 ± 1.15 d
Root fresh weight/g	1.88 ± 0.28 b	2.10 ± 0.29 b	3.06 ± 0.25 a	1.30 ± 0.21 c
Shoot dry weight/g	8.00 ± 0.24 c	9.07 ± 0.52 b	9.87 ± 0.22 a	6.52 ± 0.23 d
Root dry weight/g	0.46 ± 0.06 b	0.51 ± 0.06 b	0.69 ± 0.04 a	0.33 ± 0.03 c
Root-to-shoot ratio	5.79 ± 0.91 b	5.64 ± 0.80 b	6.98 ± 0.45 a	5.08 ± 0.59 b

^1^ Values are mean ± standard error (SE) from three independent replicates (*n* = 3). Different lowercase letters within the same row indicate significant differences at *p* < 0.05.

**Table 2:** Effects of different P levels on photosynthetic parameters of non-jointed water dropwort.

Parameter	CK	LP	MP	HP
Chlorophyll a/mg g^−1^	1.45 ± 0.21 b	1.62 ± 0.14 ab	1.75 ± 0.06 a	1.22 ± 0.09 c
Chlorophyll b/mg g^−1^	0.64 ± 0.05 bc	0.66 ± 0.08 b	0.76 ± 0.05 a	0.57 ± 0.02 c
Total chlorophyll/mg g^−1^	2.09 ± 0.25 b	2.29 ± 0.12 ab	2.51 ± 0.11 a	1.79 ± 0.09 c
Chlorophyll a/b	2.29 ± 0.28 a	2.49 ± 0.44 a	2.31 ± 0.08 a	2.16 ± 0.17 a
Carotenoids/mg g^−1^	0.25 ± 0.04 c	0.37 ± 0.03 b	0.46 ± 0.03 a	0.23 ± 0.04 c
Pn/μmol·m^−2^·s^−1^	8.43 ± 0.31 b	9.60 ± 0.37 a	0.68 ± 0.04 a	0.36 ± 0.06 c
Gs/mol m^−2^ s^−1^	0.56 ± 0.03 b	0.60 ± 0.05 b	0.68 ± 0.04 a	0.36 ± 0.06 c
Ci/μmol·mol^−1^	338.72 ± 1.72 b	339.21 ± 1.68 b	334.86 ± 1.45 b	358.95 ± 2.24 a
Tr/mmol·m^−2^·s^−1^	3.85 ± 0.45 b	3.83 ± 0.31 b	4.27 ± 0.25 a	2.90 ± 0.16 c

Values are mean ± standard error (SE) from three independent replicates (*n* = 3). Different lowercase letters within the same row indicate significant differences at *p* < 0.05.

## Data Availability

The original contributions presented in this study are included in the article and Supplementary Material. Further inquiries can be directed to the corresponding authors.

## Supplementary Materials

The following supporting information can be downloaded at https://www.mdpi.com/article/10.3390/metabo16020101/s1. Figure S1: Phenotypic comparison of non-jointed water dropwort under different phosphorus treatments. Figure S2: TIC chromatograms of non-jointed water dropwort root samples under different P treatments in positive and negative ion modes; Figure S3: Multivariate statistical analysis of metabolites in CK and HP groups; Figure S4: OPLS-DA model permutation test analysis; Table S1: Ten significantly differential metabolites in non-jointed water dropwort under high P stress.
