# Supplementary material for: Mechanistic Analysis of Physiological and Metabolic Responses in Non-Jointed Water Dropwort Under Phosphorus Stress
Source: Metabolites. 2026 Jan 29;16(2):101. doi: 10.3390/metabo16020101 (PMC12943298; doi:10.3390/metabo16020101)
Supplement: Supplementary file 1 [file metabolites-16-00101-s001.zip › metabolites-4112237-supplementary.pdf]

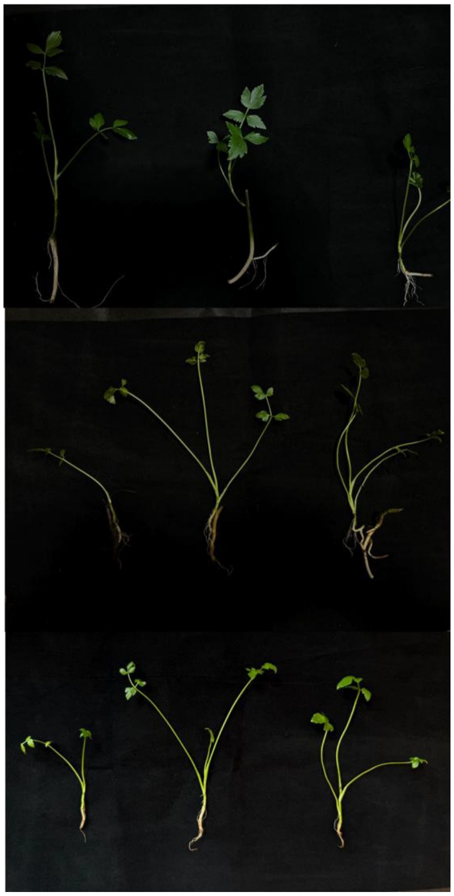

Figure S1. Phenotypic comparison of non-jointed water dropwort under different phosphorus treatments. From top to bottom: LP (low phosphorus, 5 mg·L^{-1}), MP (moderate phosphorus, 10 mg·L^{-1}), and HP (high phosphorus, 30 mg·L^{-1}).

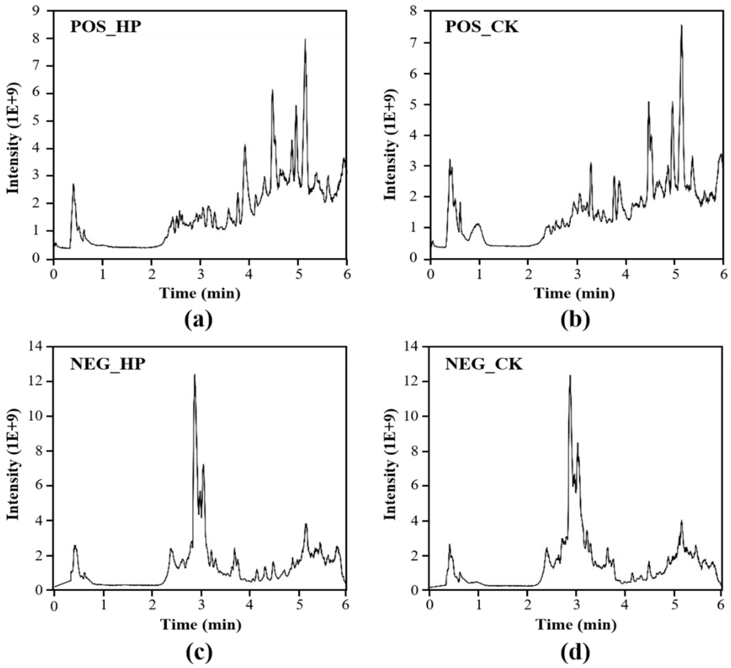

Figure S2. TIC chromatograms of non-jointed water dropwort root samples under different P treatments in positive and negative ion modes: (**a**) positive ion mode, HP group; (**b**) positive ion mode, CK group; (**c**) negative ion mode, HP group; (**d**) negative ion mode, CK group.

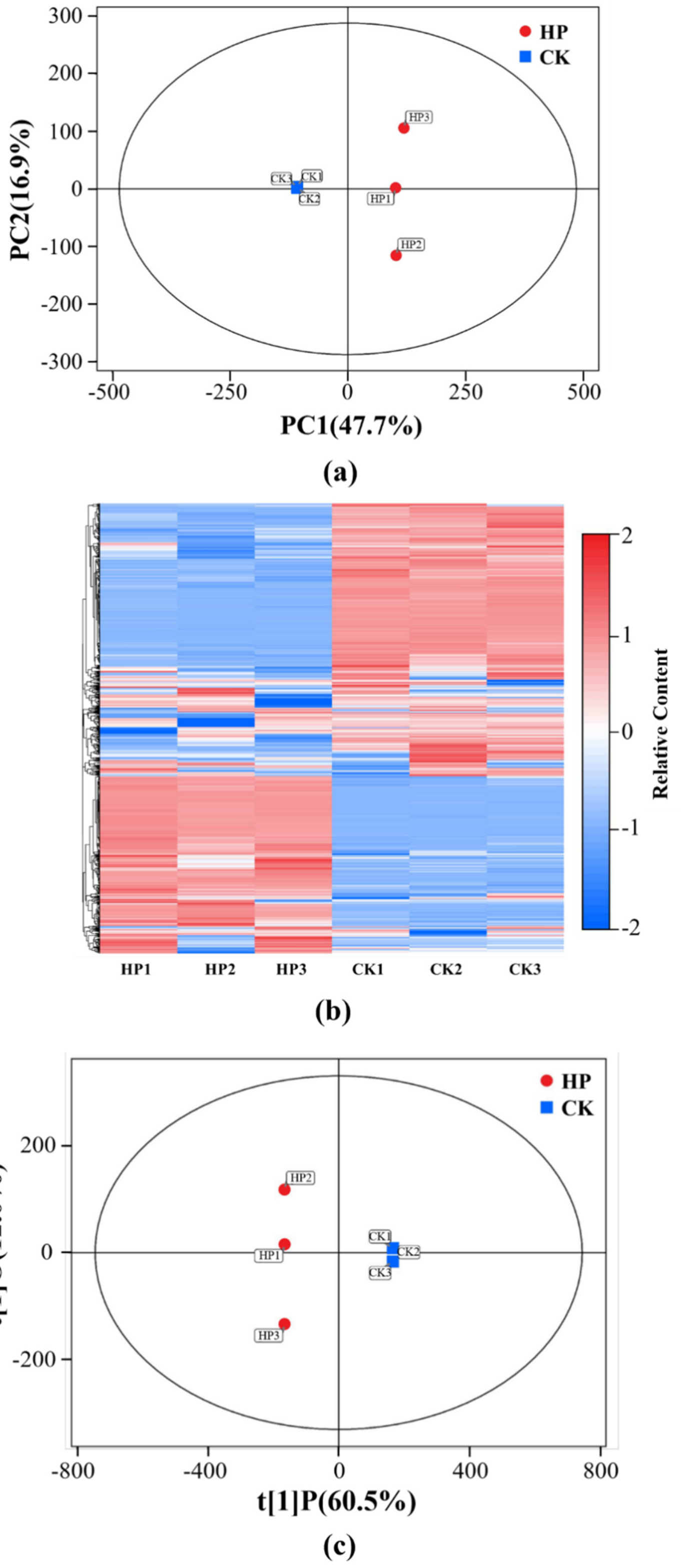

Figure S3. Multivariate statistical analysis of metabolites in CK and HP groups: (a) PCA score plot showing sample distribution based on metabolite profiles; (b) Hierarchical clustering heatmap illustrating relative metabolite abundance; (c) OPLS-DA score plot indicating group separation and model discrimination.

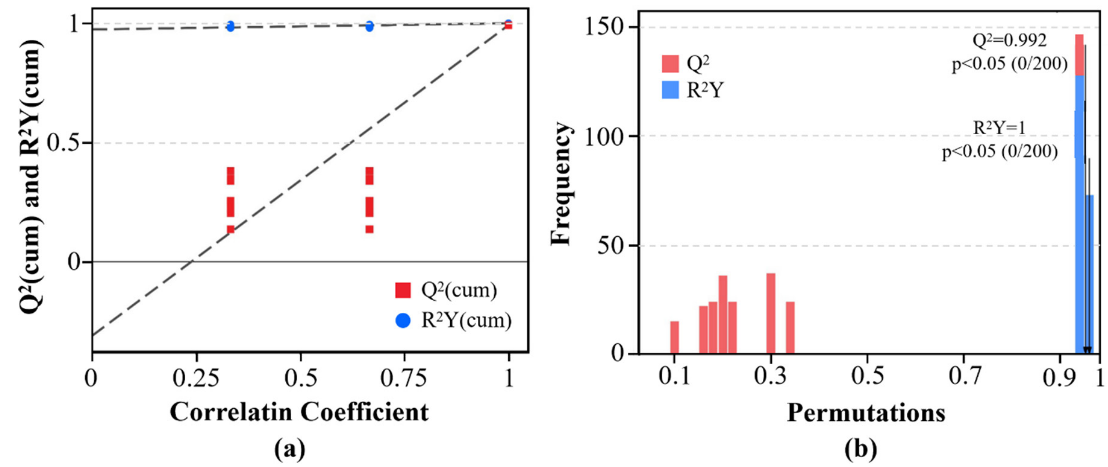

Figure S4. OPLS-DA model permutation test analysis: (a) dot plot; (b) bar plot.

Table S1. Ten significantly differential metabolites in non-jointed water dropwort under high P stress.

Name	Expression	FC	Number	Class
Narcissin	Up	37.50	1.285	Flavonoids
5,7-dihydroxy-2-(4-hydroxy-3-methoxy-phenyl)-3-[3,4,5-trihydroxy-6-[[(2R,3R,4R,5R,6S)-3,4,5-trihydroxy-6-methyl-tetrahydropyran-2-yl]oxymethyl]tetrahydropyran-2-yl]oxy-chromen-4-one	Up	37.50	1.285	Flavonoids
Isorhamnetin-3-O-neohespeidoside	Up	37.50	1.285	Flavonoids
2-(3,4-dihydroxyphenyl)-5-hydroxy-7-methoxy-3-[(2S,3R,4S,5S,6R)-3,4,5-trihydroxy-6-[[(2R,3R,4R,5R,6S)-3,4,5-trihydroxy-6-methyl-tetrahydropyran-2-yl]oxymethyl]tetrahydropyran-2-yl]oxy-chromen-4-one	Up	37.50	1.285	Flavonoids
Quercetin_3-O-malonylglucoside	Up	39.93	1.279	Flavonoids
(3aR,6R,7aR)-6-methyl-3-methylidene-6-(4-oxopentyl)-3a,4,7,7a-tetrahydro-1-benzofuran-2,5-dione	Up	3.903	1.285	Sesquiterpenoids
Rhodiosin	Up	15.34	1.285	Flavonoids
Dioncophyllinol_B	Up	2.099	1.285	Diterpenoids
Primin	Up	1.899	1.285	Aromatic polyketides
8-hydroxy-3-methyl-isochroman-1-one	Up	78.17	1.282	Coumarins
Isoscopoletin	Down	0.277	1.285	Coumarins
Nonaethylene glycol	Down	0.305	1.285	Glycerolipids
8-benzoyl-1,5,5-trimethyl-6,15-dioxatetracyclo[9.3.1.0]pentadeca-7(12),8,10-trien-9-ol	Down	0.159	1.285	Phloroglucinols
Mitragynine	Down	0.546	1.285	Tryptophan alkaloids
2-(3,4-dihydroxyphenyl)-5,7-dihydroxy-6,8-dimethoxy-chromen-4-one	Down	0.068	1.283	Flavonoids
Axillarin	Down	0.068	1.283	Flavonoids
Tetradecasphinganine	Down	0.328	1.285	Sphingolipids
2-Methylphenol	Down	0.228	1.284	Phenolic acids
3-Cresol	Down	0.228	1.284	Phenolic acids
2-Oxo-8-methylthiooctanoic acid	Down	0.333	1.285	Fatty acids and conjugates
